# Long-Range Displacement Meters Based on Chipped Circular Patch Antenna

**DOI:** 10.3390/s20174884

**Published:** 2020-08-28

**Authors:** Songtao Xue, Kang Jiang, Shuai Guan, Liyu Xie, Guochun Wan, Chunfeng Wan

**Affiliations:** 1 Department of Disaster Mitigation for Structures, Tongji University, Shanghai 200092, China; xue@tongji.edu.cn (S.X.); 1932509@tongji.edu.cn (K.J.); tjguan@tongji.edu.cn (S.G.); 2 Department of Architecture, Tohoku Institute of Technology, Sendai 982-8577, Japan; 3 Department of Electronic Science and Technology, Tongji University, Shanghai 200092, China; wanguochun@tongji.edu.cn; 4 Key Laboratory of Concrete and Pre-Stressed Concrete Structure of Ministry of Education, Southeast University, Nanjing 210096, China; wan@seu.edu.cn

**Keywords:** displacement sensor, circular patch antenna, resonant frequency, sloping channel

## Abstract

This paper presents a passive wireless long-range displacement sensor that is based on the circular patch antenna, and the detecting range of the sensor can be customized. The sensor consists of a chipped circular antenna with two opened rectangular windows, a substrate, and a ground plate with a sloping channel. No bonding between the antenna and the ground plate allows for the chipped antenna to slide along the sloping channel. The channel will drive the current flow on the plate once the chip is activated, increasing the effective electrical length and, consequently, decreasing the resonant frequency of the circular antenna. The sensing mechanism equates the measuring displacement to the relative movement of the antenna with respect to the ground that achieves the measurement of long-range displacement and, thus, the proposed sensor can avoid stress damage to the antenna due to excessive deformation. Three different range sensors were simulated in the the Ansoft high frequency structure simulator (HFSS). The results show that the resonance frequency of the antenna has a linear relationship with the varying chute depth beneath the chip. Three sensors were fabricated, and the experimental results also validated that the sensitivity of the sensor can be adjusted.

## 1. Introduction

Nowadays, the increased use of many civil structures has increased the aging effects and sometimes lead to damage [1]. Moreover, civil structures can experience many adverse load conditions, such as earthquakes, storms, and blasts [2]. Thus, with increasing attention paid to the safety of structures, structural health monitoring (SHM) has seen a surge of interest in recent years.

SHM is a process that works on monitoring and transmitting the information of the structure related to its current condition, such as deformations, stresses, and responses. In SHM, sensors are used for periodic and continuous monitoring, which not only detect subtle deformations but also record the formation of cracks [3]. With this type of information, engineers can evaluate the safety of a structure and make detailed maintenance plans. Generally, sensors are classified as wired or wireless. For wired sensors, the accuracy and resolution of the measurements are high, but installation time and cost are also high [4]. Moreover, wired sensors have been known to stop functioning and they cannot transmit information after an earthquake, since electrical power systems are often damaged. Wireless sensors are attractive options due to their low cost and ease of installation [5]. Low costs enable large-scale wireless sensors to be deployed on structures; additionally, their small size and ease of installation allow the wireless sensors to be placed densely, increasing the spatial resolution of data collection and the accuracy of structural assessment [6]. Wired sensors depend on electric wiring to supply power that is unreliable, or they need batteries to operate that are limited based on the unpredictable lifetime of their batteries. On the contrary, wireless passive sensors can operate normally through energy harvesting, which further reduces costs and makes installation simple [7]. All of these advantages make passive wireless sensors more practical in applications.

In recent years, there have been many passive wireless sensor-based radio frequency identification (RFID) tags used for structural health monitoring [8,9,10,11,12,13,14,15,16,17]. Bhattacharyya et al. [18] designed an RFID displacement sensor that relates the RFID tag performance to the displacement. The displacement can be calculated by measuring the threshold and backscatter power. However, the accuracy of the measurements is easily affected by the metallic reflecting surface. Thai et al. [19] proposed a strain sensor that is composed of a rectangular patch and two open loops. Through two orthogonal open loops and parallel plate capacitance, the strains can be measured in two independent directions. However, for sensors based on the monolithic antenna, it is difficult to measure large deformations accurately because a failure of the antenna may occur under large deformation conditions. Meanwhile, the uncertain strain transfer ratio, stress concentration, the random formation of cracks, and insufficient bonding strength also affect the accuracy of the measurement [20]. 

The above-mentioned drawbacks can be overcome by unstressed sensors that utilize separate antennas. Zhang et al. [21] designed a circular patch antenna with an open rectangular window to monitor the development of the crack. However, monitoring the crack’s formation or development has a relative bad precision because the location, length, depth, and shape of the cracks cannot be accurately forecast. Xue et al. [22] designed a crack sensor based on the patch antenna, which relates crack width to electrical length. By analyzing the shift of the resonant frequency, the crack width can be obtained. Xue et al. [23] also proposed a displacement sensor that is composed of a helical antenna and a dielectric rod antenna. When the relative displacement of the helical antenna and the dielectric rod occurs, which can be equivalent to the displacement deformation of the structure, the resonant frequency of the sensor will shift. The configuration of the helical antenna is relatively complicated and the sensor is not easily installed. 

When patch antenna sensors are applied to the monitoring of post-seismic buildings with large residual deformations, most existing monolithic-patch-antenna-based sensors cannot meet the demands because the displacement deformation of the structure is so large that it causes the excessive deformation of antennas or break the bonding. In addition, high-rise buildings experience large horizontal displacements due to wind loads; moreover, long-span bridges under long-term loads also experience large deflection deformation. Therefore, antenna-based displacement sensors should satisfy several requirements for those situation: They should have high accuracy and a long-range detection capability for large deformations, and they should be damage free to assure stability.

In this paper, the authors proposed a novel displacement sensor utilizing a circular patch antenna with two open rectangular windows to detect deformation. Based on the technology that the resonant frequency of an antenna is inversely proportional to its electrical length [24], the displacement can be obtained by monitoring the shift of the resonant frequency. When compared to traditional sensors [25], displacement sensing is not a matter of stretching the antenna itself, but of changing the relative position of the antenna with respect to the ground plane; thus, with this separate structure the novel displacement sensor can be used for large deformation sensing without stretching and bonding issues. Furthermore, thanks to the separate design, by adjusting the length of the ground plane, the measuring range of the sensor can be manipulated artificially to measure larger displacements.

This paper is presented, as follows: Section 2 presents the design principle of the displacement sensor based on the circular patch antenna. This section also introduces the rough sensor model. Section 3 describes a detailed sensor model and the numerical simulation result. Section 4 reveals the experiment design, results, and analysis of the displacement sensor. The paper ends with the conclusion and a summary of expectations based on the displacement sensor and the circular patch antenna.

## 2. Design Principle

In recent years, the circular-patch-antenna-based sensor has been introduced in SHM for deformation sensing. Daliri et al. [25] proposed a circular-patch-antenna-based sensor. By transforming the strain to the radius of the circular antenna, a linear relationship between the frequency and strain can be obtained. Another form of the circular patch antenna involves utilizing its strain sensing structure, which has several slits. Slits in an antenna can increase the effective electrical length and create a more obvious shift of frequency. Lopato et al. [26] studied the first and second frequencies of circular antennas and pointed out that the direction of load is more sensitive for the first frequency.

**Figure 1:**
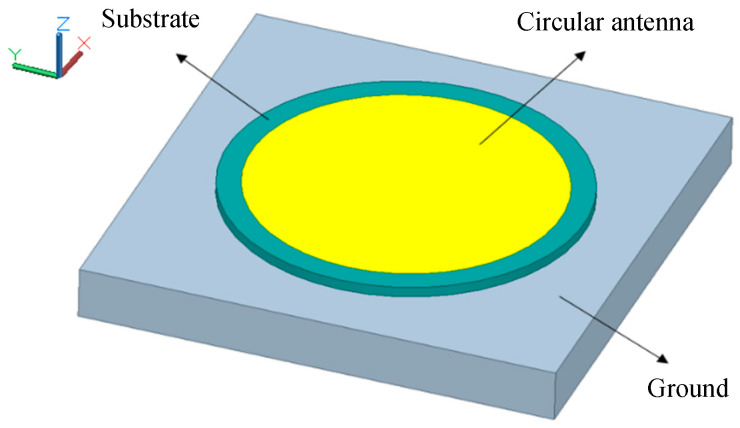
Circular antenna.

**Figure 2:**
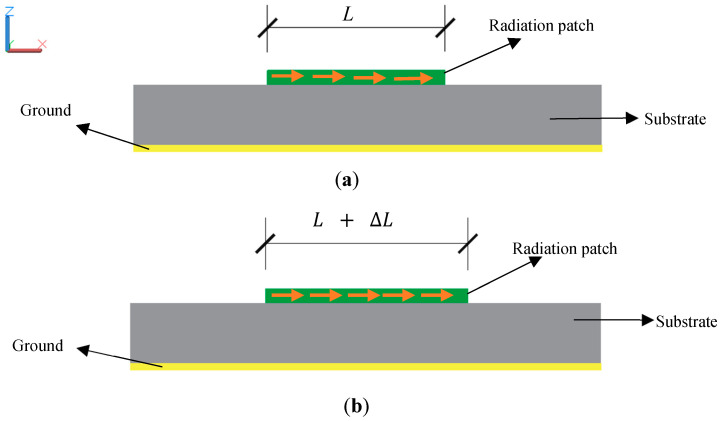
Schematic diagram of a variation in electrical length: (**a**) the initial state of the antenna and (**b**) the deformed state of the antenna.

According to the antenna theory, the circular antenna that is shown in Figure 1 can be simplified to a resonant cavity to solve the electric field, and the size of the resonator cavity affects the resonant frequency [27]. While monitoring the shift of frequencies, we can calculate the change of physical dimensions and obtain the deformation information. Meanwhile, the effective electrical length can be artificially increased by changing the physical length and electrical specification of the transmission lines, which will also cause the resonant frequency of the antenna to shift, as illustrated in Figure 2. Through this technology, the sensor can relate the frequency to a change in physical length to achieve displacement sensing.

### 2.1. Design of Displacement Sensor

**Figure 3:**
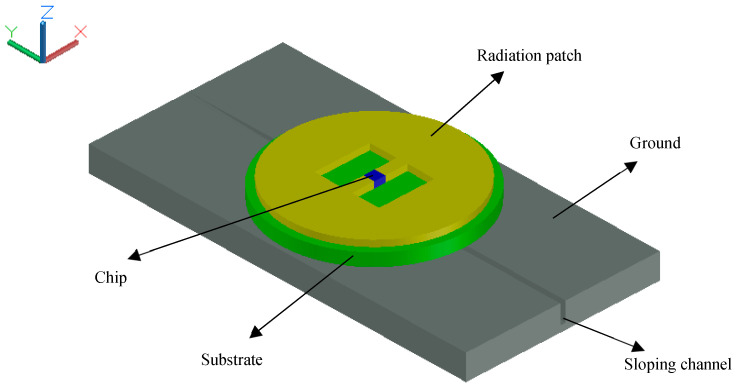
Displacement sensor.

Based on the above theory, a displacement sensor is proposed in this paper, as shown in Figure 3. The sensor is composed of a radiation circular patch, chip, substrate, and ground. The radiation patch with a chip is bonded with the substrate, and the substrate is able to slide on the top of the ground without bonding. There is a sloping channel with deepening rectangular section cut in the upper surface of the aluminum plate. Two rectangular windows are opened in the circular patch of the antenna, which changes the current path on the radiation patch and causes more current to flow on the copper sheet between the two rectangular windows, correspondingly increasing the sensitivity of the displacement sensor. Once the chip is activated, the electric current will flow within the circular antenna and the surface of the ground and the sloping channel.

**Figure 4:**
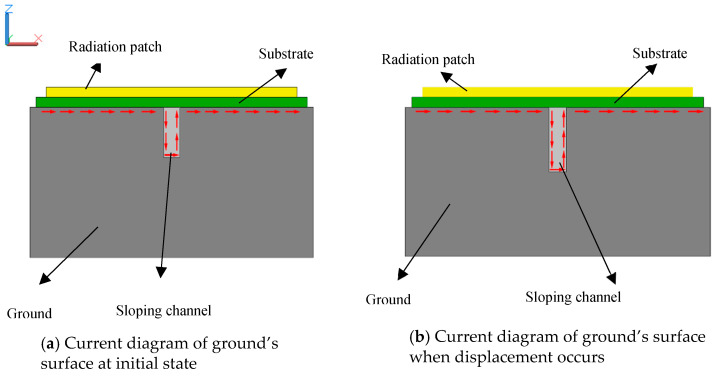
Current diagram of ground’s surface.

In practical engineering applications, the circular patch antenna and ground are fixed onto two different components in a structure, respectively, for instance, each side of a crack. While the relative displacement between the two components that increases the chip’s underlying chute depth takes place, the ground and the antenna produce relative movement. The movement changes the electrical length of the surface of the ground and the chute, as shown in Figure 4. The channel will drive the current flow on the plate, increasing the effective electrical length, and, consequently, decreasing the resonant frequency of the circular antenna. Subsequently, the variation of the resonant frequency of the antenna can be detected by the reader that can be transformed into the information of displacement.

### 2.2. The Sensing Principle of Circular Antenna

For the sensor presented in this paper, the resonant frequency will be shifted with the increase of the effective electrical length. The electrical length is the ratio of the physical length of the transmission line to the wavelength of the electromagnetic wave transmitted on the line. The electrical length can also be expressed as the physical length of the transmission line times the ratio of the transmission time of an electrical or electromagnetic signal in a medium of a given length to the time that it takes for the signal to pass the same length in free space [28]:

(1) $$L_{e}=L\times \frac{t_{1}}{t_{2}},$$

where $L_{e}$ is the electrical length of the transmission line, L is the physical length of the transmission line, $t_{1}$ is the transmission time of an electrical or electromagnetic signal in a medium of a given length, and $t_{2}$ is the time that it takes for the signal to pass the same length in free space. 

$t_{1}$ and $t_{2}$ are calculated by:

(2) $$t_{1}=L/v_{p},$$

(3) $$t_{2}=L/c,$$

where $v_{p}=c/\sqrt{\varepsilon_{eff}}$, $v_{p}$ is the speed at which an electrical or electromagnetic signal travels through a medium and $\varepsilon_{eff}$ is the effective dielectric constant.

Thus, $L_{e}$ can be calculated as:

(4) $$L_{e}=L\times \sqrt{\varepsilon_{eff}},$$

For a certain physical medium, its electrical length is always greater than its physical length. For example, the electrical length of the coaxial line is greater than the physical length of the coaxial line due to the existence of resistance, capacitance, and inductance, which hinder the transmission of internal signals.

According to the electromagnetic field distribution of the coaxial line, the capacitance per unit length can be expressed as [27,29]:

(5) $$C=\varepsilon_{r}/18\ln(D/d),$$

where D is the inner diameter of the coaxial outer conductor and d is the outer diameter of the inner conductor [29].

The coaxial impedance component independent of frequency is given by [29]:

(6) $$Z_{\infty }=\frac{60}{\sqrt{\varepsilon_{r}}}\ln\frac{D}{d},$$

Using Equation (5) and Equation (6), $\varepsilon_{r}$ can be obtained as:

(7) $$\sqrt{\varepsilon_{r}}=\frac{3}{10}Z_{\infty }C_{e},$$

where $C_{e}$ is the capacitance of a coaxial transmission, $C_{e}=CL$, and $\varepsilon_{r}$ is the relative dielectric constant.

The relative dielectric constant can also be expressed as [29]:

(8) $$\varepsilon_{r}=\varepsilon /\varepsilon_{o},$$

where ε is the dielectric constant and $\varepsilon_{o}$ is the dielectric constant of vacuum.

For the coaxial line, the dielectric constant is approximately equal to the effective dielectric constant. Thus, the electrical and physical length of the transmission line are respectively expressed as:

(9) $$L_{e}=\frac{3}{10}Z_{\infty }CL=\frac{3}{10}Z_{\infty }C_{e},$$

(10) $$L=\frac{3}{10}\frac{1}{\sqrt{\varepsilon_{eff}}}Z_{\infty }CL=\frac{3}{10}\frac{1}{\sqrt{\varepsilon_{eff}}}Z_{\infty }C_{e},$$

The impedance calculated by Equation (6) is not accurate, because D/d and $\varepsilon_{r}$ are uncertain. In actual measurements, $Z_{\infty }$ is usually calculated as:

(11) $$Z_{CR}=Z_{\infty }+A/\sqrt{f},$$

where A is the parameter of the coaxial line itself, which reflects the loss of electromagnetic wave energy when transmitting along the cable, $Z_{CR}$ is the real part of the coaxial impedance, which can be measured by the resonance method. Thus, $Z_{CR}$ is calculated as:

(12) $$Z_{CR}=n/4C_{e}f,$$

where n is the harmonic number and f is the frequency corresponding to n.

The following formula can be obtained from Equation (10):

(13) $$\frac{\Delta L}{L}=-\frac{\Delta \sqrt{\varepsilon_{eff}}}{\sqrt{\varepsilon_{eff}}}+\frac{\Delta Z_{\infty }}{Z_{\infty }}+\frac{\Delta C_{e}}{C_{e}},$$

The following formula can be obtained from Equation (11) and Equation (12):

(14) $$\frac{\Delta Z_{\infty }}{Z_{\infty }}=-\frac{Z_{CR}}{Z_{\infty }}\frac{\Delta C_{e}}{C_{e}}-\frac{Z_{CR}-A/2\sqrt{f}}{Z_{CR}}\frac{\Delta f}{f},$$

Because A is the performance parameter of the coaxial line itself, the above equation can be simplified to:

(15) $$\frac{\Delta Z_{\infty }}{Z_{\infty }}\approx -\frac{\Delta C_{e}}{C_{e}}-\frac{\Delta f}{f},$$

Substituting Equation (15) into Equation (13), we can obtain:

(16) $$\frac{\Delta L}{L}=-\frac{\Delta \sqrt{\varepsilon_{eff}}}{\sqrt{\varepsilon_{eff}}}-\frac{\Delta f}{f}$$

For electrical length, the formula can be simplified to:

(17) $$\frac{\Delta L_{e}}{L_{e}}=-\frac{\Delta f}{f},$$

Clearly, the frequency will decrease with the increasing electrical length. For circular patch antenna, the radiation characteristics are similar to resonant monopole and the fringing fields are symmetrical around the circumference, which are equivalent to a coaxial line [30]. Equation (17) can be applied to circular antennas. The finite element software high frequency structure simulator (HFSS) is used to simulate three sensors with different ranges to examine the working performance of the sensors. Section 3 describes detailed dimension parameters and simulation results.

## 3. Modeling and Simulation

**Figure 5:**
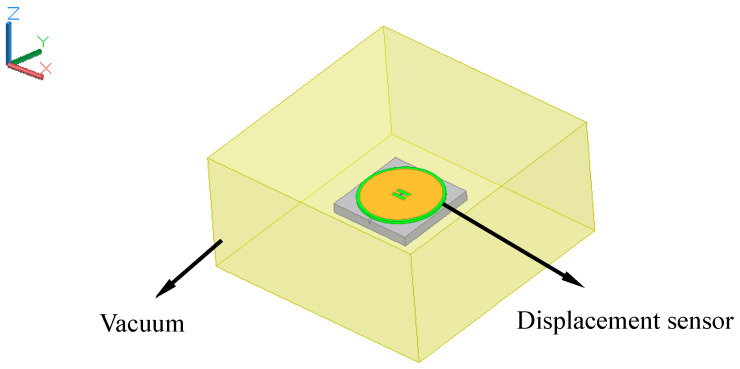
Displacement sensor model using Ansoft high frequency structure simulator (HFSS).

The finite element model of the displacement sensor was simulated in Ansoft HFSS. Figure 5 shows this model, which consists of an upper radiation patch, substrate, and ground plane. The upper radiation patch is made of copper. The material of the substrate was selected during the design process as FR4-epoxy, and the chosen ground plane is an aluminum plate with a chute. The connection between the upper patch and the substrate, the substrate, and the aluminum plate is seamless and in close contact. The circular patch antenna is placed inside a vacuum chamber and the vacuum cavity is set as the radiation boundary condition. The size of the vacuum chamber is 200 mm × 200 mm × 100 mm, which can ensure that the distance between the circular patch antenna and surface of the vacuum chamber is larger than one-quarter of the wavelength corresponding to the fundamental resonant frequency of the circular patch antenna. The boundary condition of the upper radiation patch is set as perfect E, so the electric field vector is perpendicular to the surface. The excitation mode is set to a lumped port excitation.

**Figure 6:**
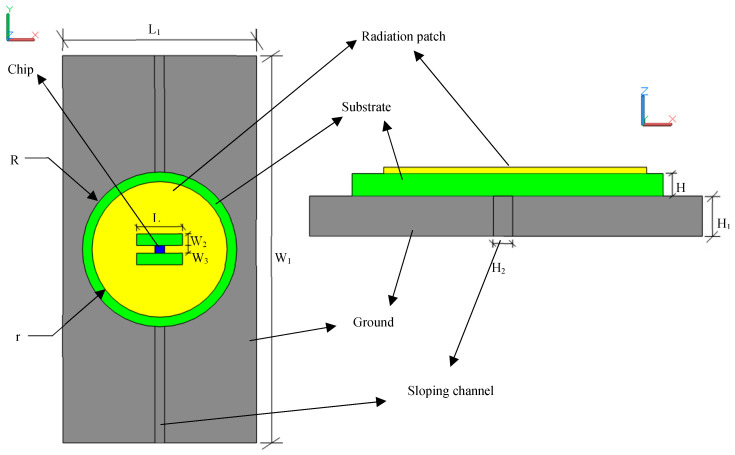
Diagram of the sensor’s specific parameters.

By adjusting the diameter of the radiation patch and the size of the rectangular window, a suitable size of the circular patch antenna can be found to meet the requirement of impedance matching. Table 1 lists the detailed parameters of the sensor and Figure 6 diagrams specific parameters that are important to this discussion.

In the HFSS model, the depth of the chute is taken as a variable for the convenience and operation of the model. As previously mentioned, the sensor is designed to monitor the displacement. Therefore, in data processing, change in the depth of the aluminum plate’s chute needs to be converted into the relative displacement between the aluminum plate and the substrate. Three aluminum plates with different chute slopes were designed at 1:70, 2:70, and 3:70 (the ratio between the depth difference and the slope length) to simulate the performance of the displacement sensors with different size of chutes. To verify that the range is customizable, three range displacement sensors were also designed at 70 mm, 135 mm, and 280 mm (the difference between the length of the aluminum plate and the diameter of the substrate).

**Figure 7:**
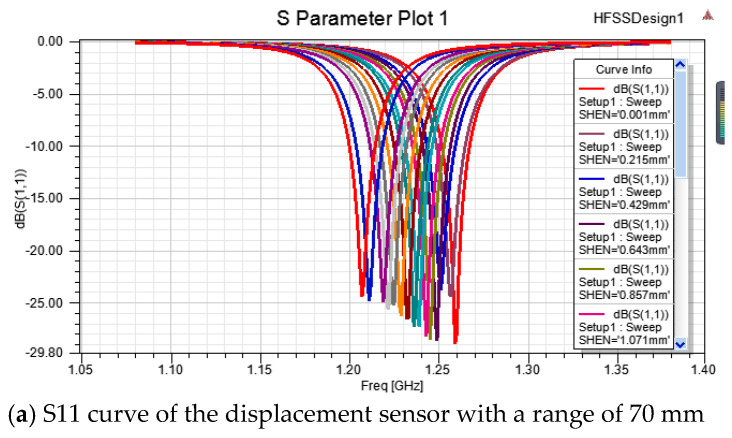

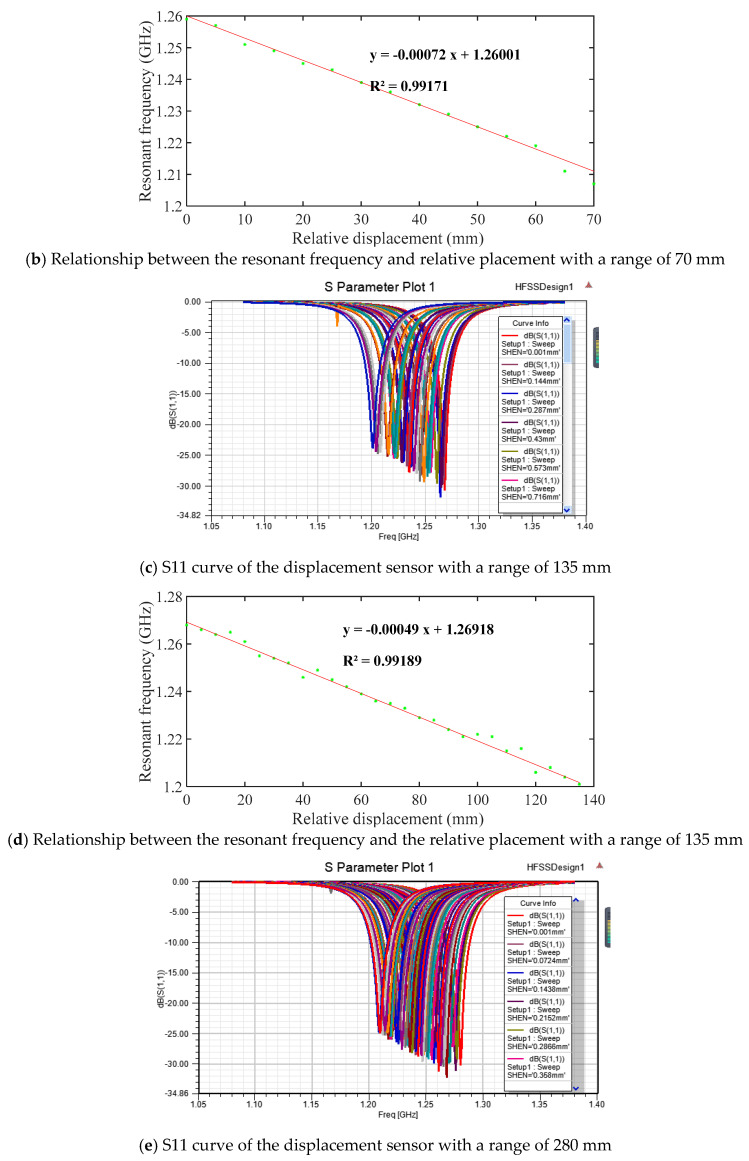

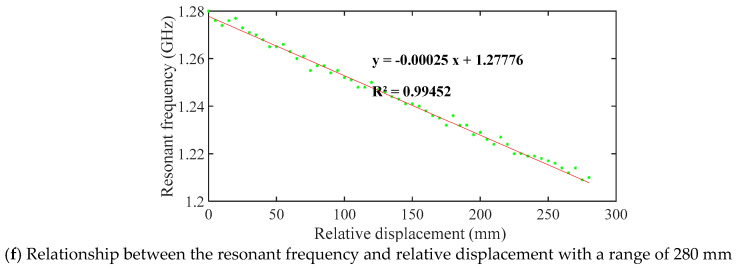
Simulation results of three types of displacement sensors.

When the relative displacement increases the chip’s underlying chute depth, the resonant frequency of the antenna decreases gradually, as shown in Figure 7. This is consistent with theoretical analysis showing that resonant frequency will decrease as the increase of the electrical length.

For displacement sensors with ranges of 280 mm, 135 mm, and 70 mm, the linear correlation coefficients are 0.99171, 0.99189, and 0.99452, respectively. This shows that the resonance frequency of the antenna has a linear relation with the relative displacement, and the linear fitting effect is good.

**Figure 8:**
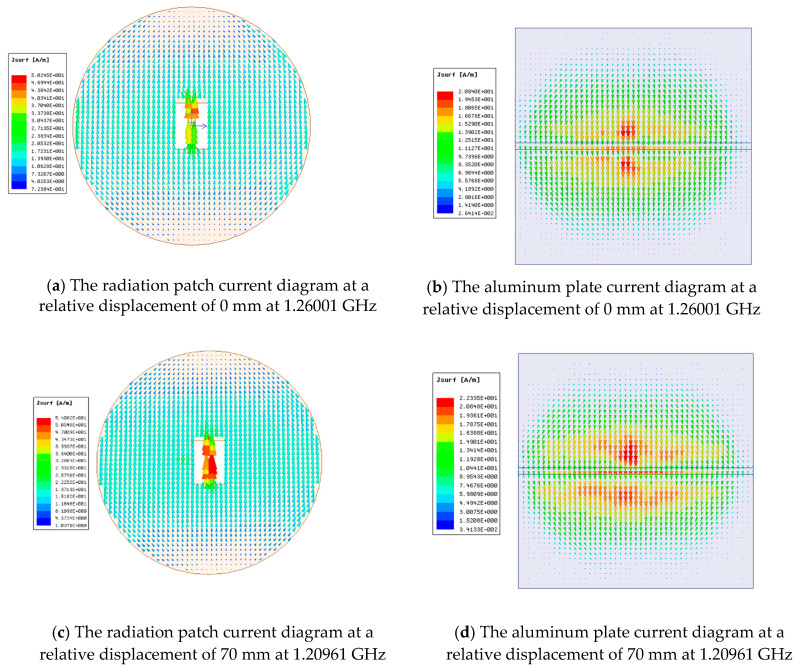
Current diagram of the radiation patch and aluminum plate.

**Figure 9:**
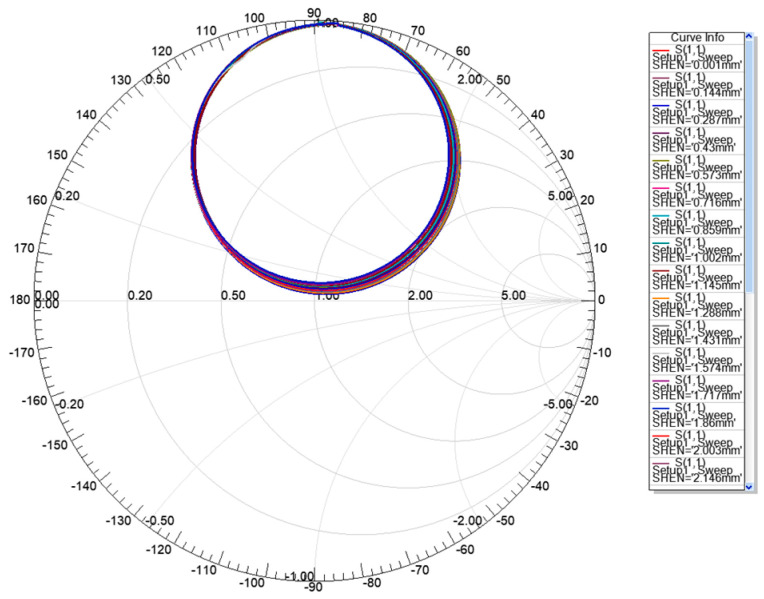
Smith chart of the displacement sensor at 1.21 GHz.

**Figure 10:**
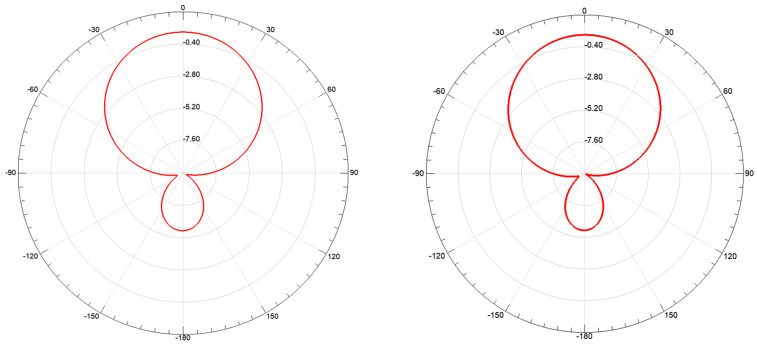
Radiation pattern diagram at 1.21 GHz: xz-plane (**left**) and yz-plane (**right**).

Generally, the existence of the chute will disturb the current distribution of the radiation patch and the ground and the field distribution in the substrate. This disturbance will affect the radiation characteristics of the antenna and the energy that is stored in the antenna, thereby affecting the impedance of the antenna. Figure 8 shows the current diagram of the radiation patch and the aluminum plate. It can be seen that, while the relative displacement of the substrate and the aluminum plate that increases the chip’s underlying chute depth occurs, the depth of the chute at the bottom of the substrate will change, and the current diagram of the radiation patch and aluminum plate also changes, which helps to increase the effective electrical length of the current on the metal surface and, thus, decreases the resonant frequency of the antenna. Figure 9 illustrates the variation of Smith charts with the depth of chute, which shows that the impedance loop moves toward the upper part in the Smith chart, indicating that input resistance decrease with the increase of the depth of chute. Figure 10 shows radiation pattern diagram of the antenna. It can be seen that the antenna has good working ability in the area above the radiation patch.

## 4. Experiment

Three sensors with the same range as those in Section 3 were tested in order to verify simulation results. The upper radiation patch was made of copper. When considering the cost of the circular patch antenna, FR4-epoxy was selected as the middle substrate of the upper radiation patch. The design also features three aluminum plates with different lengths and a chuteslope.

### 4.1. Experimental Design

**Figure 11:**
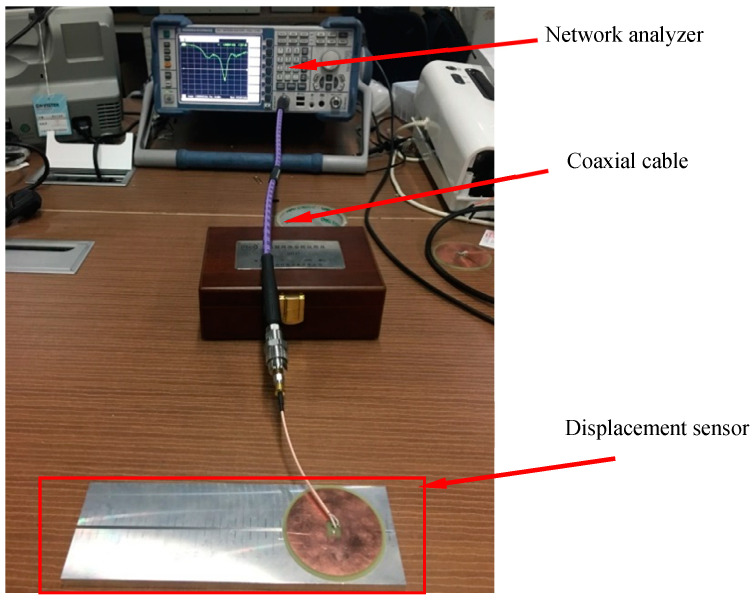
Measuring resonant frequency by using Vector Network Analyzer.

In this experiment, a vector network analyzer was used to measure the resonant frequency of the circular patch antenna. The circular antenna was connected to the vector network analyzer through the coaxial line. The circular patch antenna, substrate and aluminum plate constitute the displacement sensor together. Figure 11 shows the experimental setup.

**Figure 12:**
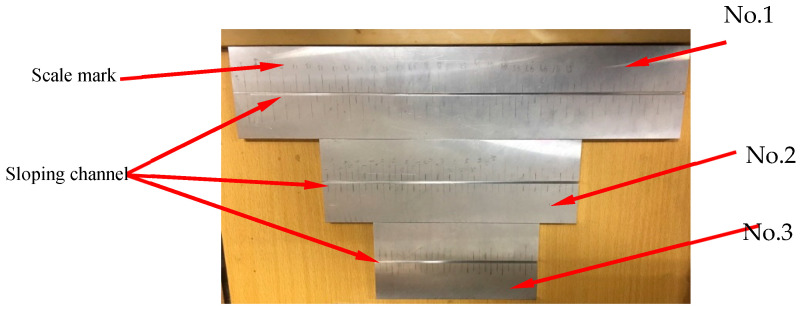
Aluminum plate with sloping channel.

Three different lengths of the aluminum plates were fabricated to verify the customizable range of the displacement sensor based on the circular patch antenna: 350 mm, 210 mm, and 140 mm. The three aluminum plates are labeled No. 1, No. 2, and No. 3 from long to short. Chute of each aluminum plate has a depth of 0 mm at its shallowest part and 6 mm at its deepest part. Thus, the slopes of the three aluminum plates are 1:70, 2:70, and 3:70. The measuring ranges of aluminum plates are 280 mm, 135 mm, and 70 mm, respectively. Figure 12 shows the aluminum plates.

The direction in which the substrate moves along the shallow part of the chute to the depth of the chute is defined as the direction in which the substrate advances. In the experiment, we had to make sure that the substrate was kept close to the surface of the aluminum plate, and the central position of the substrate had to be flush with the central position of the sloping channel; moreover, the substrate could not be skewed or reversed. Subsequently, the displacement sensor was able to adopt the first-order frequency of the antenna in order to measure the relative displacement of the structure.

The experimental process is as follows: first, the substrate and the circular antenna are placed on the aluminum plate. The outer edge of the substrate is tangent to the short edge of the aluminum plate, and the relative displacement of the substrate and aluminum plate is 0. Subsequently, the substrate moves forward 5 mm for each step, and the relative electrical length of the antenna increases. Record the $S_{11}$ curve of the antenna and the resonant frequency of the circular patch antenna is the lowest point of the curve. Section 4.2 describes the variation trend of the resonant frequency.

### 4.2. Experimental Results and Discussion

**Figure 13:**
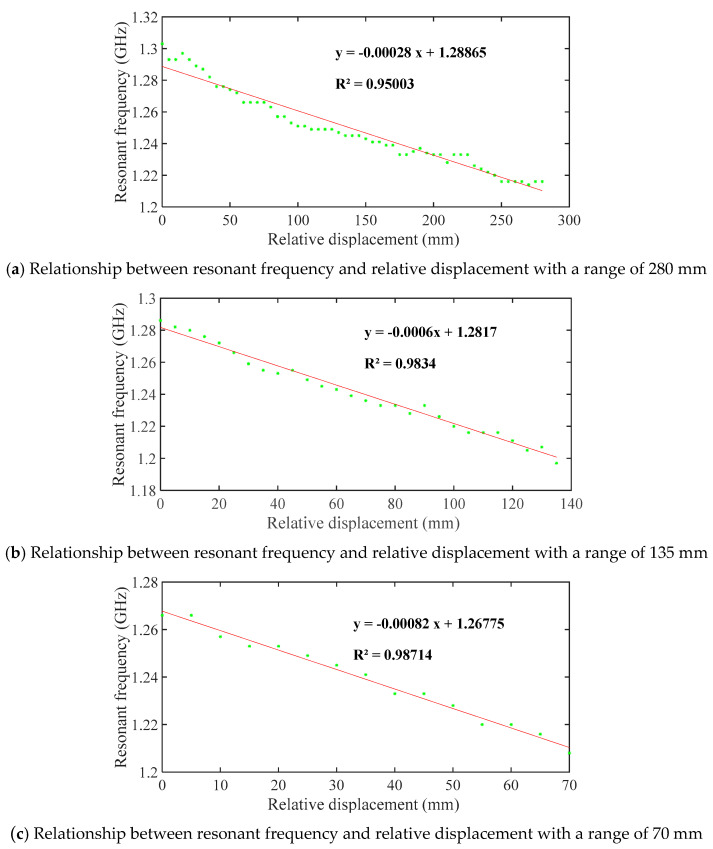
Relationships between resonant frequency and relative displacement at different ranges.

Based on the experimental results, the resonant frequency is extracted, and Figure 13 shows the relationship between the resonant frequency and the relative displacement of three displacement sensors at different ranges. Table 2 compares the sensitivity and the initial resonant frequency of the simulation results and experimental results. With the increase of relative displacement that increases the chip’s underlying chute depth, the first resonant frequency of the displacement sensor decreases. A good linear relationship exists between the resonant frequency and the relative displacement, and the correlation coefficients of the fitted line representing the three displacement sensors are all greater than 0.95.

The sensitivity and the ranges of the displacement sensors are different for three aluminum plates with different size chute. For the displacement sensor with ranges of 280 mm, 135 mm, and 70 mm, the resonant frequency offsets are 87 MHz, 89 MHz, and 59 MHz, respectively, and the sensitivities are 0.311 MHz/mm, 0.659 MHz/mm, and 0.843 MHz/mm, respectively. The sensitivity formula is expressed as:

(18) $$s=\frac{\Delta f}{W_{1}}=\frac{f}{L_{e}}\frac{\Delta L_{e}}{W_{1}},$$

where s is the sensitivity of the sensor and $W_{1}$ is the measuring range of the sensor.

For the above three sensors with different ranges, their initial resonant frequency and initial electrical length are the same, so the sensitivity is only related to ∆*L_e_*/*W*_1_, which is equal to the chute slope. Clearly, the sensitivity ratios of the experiment, simulation, and theory of the three sensors are very close.

As the slope of the chute increases, the sensitivity of the displacement sensor will increase, but the range of the displacement sensor will be reduced. The resonance frequencies of the experiment and the simulation are very close, indicating the accuracy of the simulation.

Table 3 summarizes the performances of several different types of sensors. When compared to them, the sensitivity of the sensor proposed in this paper is moderate and it can be adjusted to meet actual requirements; the measuring range is obviously larger than other sensors and, thus, the sensor can be detected large deformation.

There are some differences between the results of the experiment and the simulation, especially the sensitivity. These differences may be caused by the following effects:

(1) Effect of device: the quality of the welded joint between the coaxial line and the antenna can affect the impedance. Moreover, a gap filled with air exists between the substrate and the aluminum plate in the experiment, which causes a difference between the results of experiment and simulation.
(2) Effect of operation: in the experiment, the central position of the substrate cannot be completely flush with the central position of the chute.

## 5. Conclusions

This paper presented a novel chipped displacement sensor based on a circular patch antenna, sliding along a sloping channel of an aluminum plate, which are able to measure long-range relative displacement on a structure. While using the theoretical analysis, the authors revealed that the deepening channel actually increase the effective electrical length and, consequently, decrease the resonant frequency of the circular antenna. The resonant frequency of the antenna has a linear relationship with the varying depth. Three prototypes were modeled in the HFSS and fabricated for the experiment. The results both show that the resonant frequency of the antenna will decrease with the increase of the chip’s underlying chute depth that is caused by the relative displacement of the chipped antenna and the aluminum plate. The correlation coefficients of the fitted line of the resonant frequency and relative displacement of the experiment and simulation results are all greater than 0.95. The experiment results also validated that the sensitivity of the sensor can be adjusted by changing the slope of the chute. The errors between experiment and simulation are due to manufacturing error and experimental effect. In the following research, a smaller size of the circular patch antenna and higher precision of the displacement sensor can be pursued through theoretical research and numerical simulation.

## Figures and Tables

**Table 1:** Optimal dimension of the model (Unit: mm)

Parameters	L	$L_{1}$	R	r	$W_{2}$	$W_{3}$	H	$H_{1}$	$H_{2}$
Dimensions	12.0	70.0	33.5	31.0	3.5	2.0	2.0	8.0	2.0

**Table 2:** Sensor experimental results.

Sensor Number	NO.1	NO.2	NO.3
Measuring range of sensor (mm)	280	135	70
Chute slope	1:70	2:70	3:70
Sensitivity of experiment (MHz/mm)	0.311	0.659	0.829
Sensitivity of simulation (MHz/mm)	0.250	0.496	0.957
Error of sensitivity (%)	19.54	24.72	−15.52
Initial resonant frequency of the experiment (GHz)	1.303	1.286	1.266
Initial resonant frequency of the simulation (GHz)	1.280	1.268	1.259
Error of the initial resonant frequency (%)	1.77	1.40	0.55

**Table 3:** Comparison of the performance of sensors.

Antenna Type	Sensitivity (MHz/mm)	Measuring Range (mm)	Refs.
Helical antenna	0.616	7	[23]
Meandered circular antenna	0.6	10+	[25]
Rectangular antenna	22.1	16+	[3]
Rectangular antenna	17	4.95+	[31]
